# Glaucoma: A Degenerative Optic Neuropathy Related to Neuroinflammation?

**DOI:** 10.3390/cells9030535

**Published:** 2020-02-25

**Authors:** Stéphane Mélik Parsadaniantz, Annabelle Réaux-le Goazigo, Anaïs Sapienza, Christophe Habas, Christophe Baudouin

**Affiliations:** 1 Sorbonne Université, INSERM, CNRS, Institut de la Vision, 17 rue Moreau, F-75012 Paris, France; annabelle.reaux@inserm.fr (A.R.-l.G.); anais.sapienza@gmail.com (A.S.); cbaudouin@15-20.fr (C.B.); 2 Service de NeuroImagerie, Centre Hospitalier National d’ophtalmologie des Quinze-Vingts, F-75012 Paris, France; chabas@15-20.fr; 3 Department of Ophthalmology, Hôpital Ambroise Pare, AP HP, F-92100 Boulogne, France; 4 CHNO des Quinze-Vingts, IHU FOReSIGHT, INSERM-DGOS CIC 1423, 28 rue de Charenton, F-75012 Paris, France; 5 Université Versailles St Quentin en Yvelines, Paris Saclay, F-78180 Montigny-Le-Bretonneux, France

**Keywords:** ocular hypertension, neuronal degeneration, neuroinflammation, central visual pathway

## Abstract

Glaucoma is one of the leading causes of irreversible blindness in the world and remains a major public health problem. To date, incomplete knowledge of this disease’s pathophysiology has resulted in current therapies (pharmaceutical or surgical) unfortunately having only a slowing effect on disease progression. Recent research suggests that glaucomatous optic neuropathy is a disease that shares common neuroinflammatory mechanisms with “classical” neurodegenerative pathologies. In addition to the death of retinal ganglion cells (RGCs), neuroinflammation appears to be a key element in the progression and spread of this disease. Indeed, early reactivity of glial cells has been observed in the retina, but also in the central visual pathways of glaucoma patients and in preclinical models of ocular hypertension. Moreover, neuronal lesions are not limited to retinal structure, but also occur in central visual pathways. This review summarizes and puts into perspective the experimental and clinical data obtained to date to highlight the need to develop neuroprotective and immunomodulatory therapies to prevent blindness in glaucoma patients.

## 1. Introduction

Glaucoma is a disease of the visual system leading to irreversible blindness that is expected to affect 80 million people worldwide by 2020 [1] and 112 million in 2040 [2]. One percent of people over 40 years of age are affected by this pathology, and the proportion rises to 5% among people over 70 and 10% of those over 80. Open-angle glaucoma (OAG) is the most common form of glaucoma. OAG is characterized by degeneration of the trabecular meshwork (TM) (the filter responsible for the drainage of aqueous humor from the eye’s anterior chamber), which increases intraocular pressure (IOP). This ocular hypertension results in the impairment of the axons of retinal ganglion cells (RGCs) forming the optical nerve, and then progressive concentric loss of the RGCs. Glaucoma is an insidious disease that remains asymptomatic for a long time, in which visual manifestations occur when there is already permanent damage. Visual manifestations are first peripheral and then central. Current treatments are mainly aimed at lowering the IOP. However, additional mechanisms other than increased IOP seem to be involved in the development and progression of this degenerative disease. Indeed, glaucoma progression can be observed in 15–25% of patients despite appropriate IOP control [3,4], and normal-tension glaucoma comprises a significant proportion of glaucoma cases in which an elevated IOP cannot explain the neurodegeneration [5].

There is accumulating evidence that glaucomatous neuropathy not only affects the retina, but also spreads to the central nervous system (CNS) [6,7,8,9,10]. Over a period of time with IOP elevation, neuronal shrinkage and neuronal loss [11,12], reduced metabolic activity [13,14], and changes in the expression patterns of several synaptic plasticity markers occur in the lateral geniculate nucleus (LGN) and visual cortex of glaucoma patients and primate models [15]. The spread of the disease in the brain may disturb visual information processing and trigger further visual field defects. Neuroinflammation in glaucoma is becoming an increasingly important component because of the role played by immune and glial cells in the early stages of the disease. Astrocytes, microglia, and infiltrating monocytes are defined as critical actors in the neuroinflammatory process in glaucoma [3,4,16,17]. This review sheds light on the neuroinflammatory mechanisms involved in the progression of glaucomatous disease. In the first part, we depict the cellular and molecular mechanisms of how immune and glial cells induce the death of RGCs. Then, we highlight the pro-inflammatory signaling pathways and attempt to demonstrate their involvement in the neurodegenerative mechanisms of the central visual structures.

## 2. Glial Reactivity and Infiltration of Immune Cells in Glaucomatous Retina

The vision loss process in glaucoma is known for being associated with alterations of functional properties and the distribution of glial cells in the retina; these changes have been identified as a state of retinal glial cell activation [18].

### 2.1. Macroglial Cell Activation

Astrocyte reactivity is initially characterized by hypertrophy of soma, hyperplasia, and, notably, an increase in the expression of glial fibrillary acidic protein (GFAP) and vimentin [19] In glaucoma patients [20,21,22] as well as in preclinical models of glaucoma, astrocyte reactivity was reported at the retina level. Müller cells (MCs) are the major glial cells in the retina. It seems that glial cells/MCs are among the first cells to respond to ocular hypertension [22]. Indeed, in a rat model of chronic intraocular hypertension, induced expression of GFAP was already observed as early as 2 h following IOP elevation [23,24,25]. Moreover, in the DBA/2J mouse, a long-lasting/chronic model of glaucoma, MC activation was detected in the retina even prior to IOP rise [26]. Finally, it was reported that glial activation in the retina occurred much earlier than the RGC loss.

Activated MCs mediate both the protective and detrimental effects on RGCs. Therefore, co-expression of GFAP and glutamine synthetase (GS) was described in the end-feet and processes of MCs after ocular hypertension [27]. Thus, in the early stage of the disease, MCs play a pivotal role in maintaining the homeostasis and integrity of the retina [28] [29,30]. They express a variety of receptors related to cell growth and survival [31,32,33]. They play a protective role by taking up glutamate (to maintain a low extracellular glutamate level and thus protect RGCs from glutamate excitotoxicity) and releasing nerve growth factor [34]. However, once overactivated, MCs release several harmful factors such as tumor necrosis factor alpha (TNFα), interleukin-1 (IL-1), and nitric oxide (NO), known to exacerbate neuronal injury [35,36]. In addition, activated glial/MCs may also affect the survival of RGCs through the activation of cell death receptors [33] and the disturbance of retinal potassium and water homeostasis [37,38]. 

A cellular component related to astrocyte activation is the existence of gap junctions between cells. The processes of astrocytes are connected via gap junctions that form a functional network, allowing the astrocytes to communicate and maintain ionic and metabolic homeostasis [39]. One theory of RGC impairment in glaucoma implicates gap junctions as propagators of the death of RGCs [40]. Connexins are the main components of gap junctions, and connexin-43 is the most abundant connexin expressed by astrocytes. Indeed, primary cultures of retinal astrocytes of normal and glaucomatous donors have shown higher expression of the gene coding for connexin-43 in the astrocytes of glaucoma patients [40]. Moreover, in the retinal cribriform plate of glaucoma patients, increased expression of connexin-43 immunoreactivity was observed in association with glial cell activation [41].

**Figure 1:**
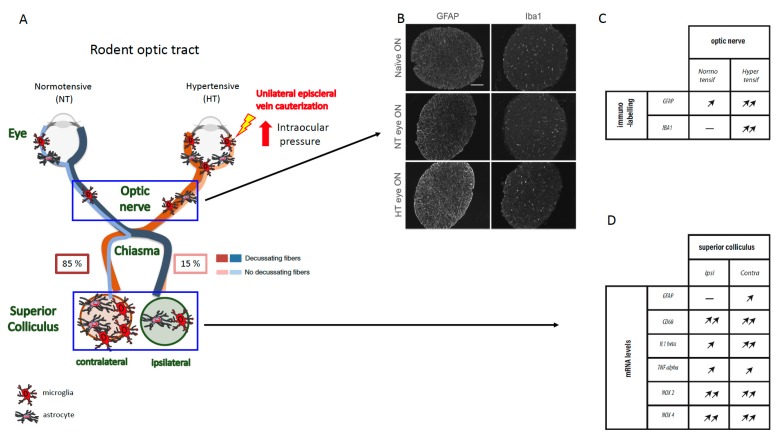
Elevated intraocular pressure (IOP) over 40 days after episcleral vein cauterization (EVC) in rat induced astrogliosis and microgliosis in the optic nerve and superior colliculus. (**A**) Diagram showing the rodent neuronal visual pathway. Immunofluorescent labeling of glial fibrillary acidic protein (GFAP) and ionized calcium binding adaptor molecule 1 (Iba1) in (**B**) naïve, contralateral (normotensive, NT), and hypertensive (HT) optic nerves. Scale bar, 100 μm. (**C**) Table of the variations of GFAP and Iba1 immunofluorescence levels in the NT and HT optic nerves, 40 days after EVC. (**D**) Table of the variations in the mRNA expression levels of inflammatory markers (i.e., GFAP, CD68, IL1β, TNFα, Nox2, and Nox 4) in ipsilateral and contralateral superior colliculus (SC), 40 days after EVC (Adapted from Sapienza et al.).

Furthermore, inflammation and astrogliosis also spread along the optic nerve after ocular hypertension. Indeed, astrocytes comprise the majority of cells in the optic nerve head. They are arranged in columns, separating the axon bundles with their processes, which are oriented perpendicular to the RGC fibers [42]. Therefore, in rodent preclinical models of glaucoma (Figure 1), higher GFAP immunostaining was found to be associated with the degeneration of (optic nerve) (ON) fibers [43,44]. 

### 2.2. Microglial Activation

Microglia are CNS-resident innate immune cells, endowed with sensor and effector functions as well as phagocytic capacity during physiological and pathological conditions [45,46,47,48]. In response to a lesion, microglia are activated, leading to morphological changes from a ramified state to an ameboid state [49], characterized by the retraction of the thinnest processes and increased soma size. The role played by reactive microglia has not yet been clearly established in glaucoma pathogenesis, but strategies aiming to inhibit reactive microglia are now being explored. At the retinal level, microglial activation and proliferation can have a harmful effect on RGCs through the secretion of pro-inflammatory cytokines such as IL-6 and TNFα or reactive oxidative species [44,46]. 

Clinically, microglial proliferation and activation were also observed in the retina and the head of the optic nerve in glaucoma patients [50,51]. In DBA/2J mice and in an ocular hypertensive model induced by episcleral vein cauterization, proliferation of microglia was reported in the retina during the disease [26]. In DBA/2J mice, microglial activation was detected beginning at three months prior to intraocular pressure rise, indicating an early event in glaucoma pathology [52]. The same holds true in a cauterized limbal-derived vein model in rats, in which the expression of the microglial marker OX42 or immunoreactivity of ionized calcium binding adaptor molecule 1 (Iba1) rises during the first two months in the optic nerve (Figure 1) [44,53]. Another study showed that the increased reactivity of microglial cells in the optic nerve appears before any sign of RGC loss in a trabecular meshwork photocoagulation model [54]. Indeed, significantly higher levels of messenger RNA (mRNA) of the *Iba1* gene, reflecting microglial proliferation, were found at the optic nerve head in this glaucoma model [52,55]. 

Collectively, these findings suggest that microglia activation is an early alteration in the retina and optic nerve during glaucoma, potentially contributing to disease onset or progression. Ultimately, the detection of microglial activation may have value in early disease diagnosis, while modulation of microglial responses may alter disease progression [52]. In this way, minocycline, a drug known to reduce microglial activation and improve neuron survival, seems to have a protective effect on RGCs in a chronic model of glaucoma (DBA/2J mice) [56]. More recently, it was shown that another antibiotic Azithromycin (with immunomodulatory properties) is able to block RGC death in retinal ischemia/reperfusion model by modifying the inflammatory state [57]. Moreover, deletion of the CD11b microglial receptor prevented microglial activation and was neuroprotective in a laser photocoagulation model [58]. In an acute model of ocular hypertension (perfusion of the anterior chamber with a hypertonic saline solution), it was shown that deletion of the Fractalkine receptor (CX3CR1) in KO mice reinforced microglial neurotoxicity and induced greater loss of RGCs [59]. These results reveal that chemokine receptor CX3CR1 modulates the activation of microglia during ocular hypertension. Thus, suppression of microglial activation seems to be a potential treatment to slow down the progression of glaucoma and improve RGC survival.

### 2.3. Transendothelial Migration of Monocytes

The role of monocyte infiltration in glaucoma pathogenesis has not yet been clearly defined. However, several preclinical studies have been conducted in this area. In DBA/2J mice, infiltration of transendothelial monocytes was detected in the retina and optic nerve at early stages of the disease [16]. However, other types of immune cells have not been found in the retinas of these animals. In addition, it was shown in this study that monocyte infiltration abrogation by a single x-ray treatment of an individual eye resulted in better RGC survival and long-term protection from glaucoma [16]. Thus, monocyte infiltration seems to be an important event in RGC death in glaucoma. As discussed above, in a laser photocoagulation model, deletion of the CD11b microglial receptor in KO mice prevented microglial/macrophagic activation and was neuroprotective [58]. However, this study did not distinguish between resident microglia and infiltrating monocytes. These data support a model of glaucomatous damage involving monocyte entry into the retina and the optic nerve; however, further investigations are needed to better understand the contribution of immune cells vs. microglia infiltrations during glaucoma progression.

Our group recently demonstrated that the increased macrophages/microglia in the retina of the hypertensive eye was correlated with an increase in CCL2 chemokine expression by astrocytes [44]. It is well known that CCL2 is strongly implicated in monocyte chemoattractivity from blood circulation to the inflammatory site [60]. Activated tissue macrophages could stem from the activation of either resident microglia or infiltrating monocytes. Tissue macrophage/microglia activation could be responsible for the increase in pro-inflammatory cytokines (TNFα and IL-1β) observed in the retina [61].

## 3. Pro-Inflammatory Signaling Pathways in Glaucoma

The induction of an inflammatory cascade in glaucoma has not yet been precisely defined. A clinical study using transcriptomic approaches to retinal and optic nerve astrocytes identified an increase in the expression of genes associated with the inflammatory pathways in glaucoma patients [62]. Therefore, an increase was shown in the expression of genes responsible for the initiation of inflammation such as the Toll-like receptor (*TLR*) and purinergic P2 receptors (P2X7), or for amplifiers of inflammation such as the TNFα gene in glaucoma patients [63,64,65].

### 3.1. Toll-Like Receptor Pathway

Analyses of glaucoma patients and experimental models of glaucoma suggest that the immune response is orchestrated in part by Toll-like receptors (TLRs). TLRs are part of innate immunity, but the recognition of pathogenic organisms is often the source of problems. During evolution, certain molecular determinants of these pathogenic organisms were selected to be clearly recognized: these are called pathogen-associated molecular patterns (PAMPs). PAMPs are specific to pathogenic organisms and therefore have no equivalent in the host (self-protection); they are structures indispensable to survival and/or the invasiveness of microorganisms. The best-known PAMPs are bacterial lipopolysaccharide (LPS) and double-stranded bacterial RNA. To date, 10 TLRs have been described in mammals (TLR1–TLR10) [66]. Increasing numbers of studies have added evidence that the oxidation products induced by glaucomatous stress could activate the glial TLRs. Anatomical studies have reported increased TLR2, TLR3, and TLR4 immunoreactive levels of microglia and astrocytes in the retinas of glaucoma patients [63]. Moreover, using a proteomics approach, a recent study evaluated the astrocyte response in a glaucoma model of the injection of hypertonic saline solution in the episclera [64]; these authors shed light on the TLR pathway and activation of nuclear factor-kappa B (NF-kB) as components of the astrocyte inflammatory response. The NF-kB pathway is indeed known to result in the expression of pro-inflammatory cytokines. Another study showed that inactivation of astroglial NF-kB reduced the inflammatory environment and improved RGC survival after retinal ischemia [67].

### 3.2. P2X7 Receptor

The P2X7 receptor, one of the purinergic P2 receptors, is activated by extracellular adenosine triphosphate (ATP) [68]. The P2X7 receptor is a ligand-gated ion channel, and its activation is induced by Na^+^ and Ca^2+^ influx [68,69]. P2X7 receptors are expressed in retinal amacrine cells, RGCs [70,71,72,73,74], microglial cells, and astrocytes [73,75] from rodent and primate glaucomatous retinas. Several studies have also demonstrated a link between P2X7R and retinal impairment. Indeed, increased ATP levels and P2X7R activation have been described in hypertensive models of glaucomatous damage in in vitro [76,77,78,79] and in vivo models [80,81,82,83,84]. In addition, this P2X7R overexpression in RGCs correlated with the loss of function through ERG measurement and with increased MAPK and caspase-3 proteins in [77,82,85] DBA/2J retinas, suggesting a pro-apoptotic mechanism in RGC death induced by high ocular pressure. Recently, it was shown in vivo that activation of P2X7 receptors is involved in the mechanism of NMDA-induced retinal injury in rats, leading to glaucomatous RGC loss [69]. 

### 3.3. TNFα Pathway

TNFα is a pro-inflammatory cytokine that is mainly synthesized by macrophages and activated T lymphocytes as well as astrocytes and microglia in the CNS. Cellular responses to TNFα are orchestrated through two distinct receptors: TNF-R1 and TNF-R2 [43,86]. More and more studies suggest that TNFα plays an important role in glaucoma disease. The inflammatory changes related to the disease were correlated with a rise of TNFα in glaucoma patients as well as in experimental models of glaucoma. Thus, a high level of TNFα was detected in the aqueous humor of OAG patients as well as in normal-pressure glaucoma patients [87]. In addition, higher levels of TNFα were found in the serum of patients with severe glaucoma compared to healthy subjects [88]. Moreover, high levels of TNFα were also found in the retinas as well as in the optic nerve astrocytes and microglia in glaucoma patients [89,90,91]. Intravitreal injections of TNFα in normotensive eyes of rabbits and mice induced axonal damage to the optic nerve comparable to that observed in glaucoma patients [58,92]. TNF-R1 and TNF-R2 deficiency resulted in the neuroprotection of RGCs in an optic nerve lesion model [93], and in a model of ocular hypertension induced by laser [58]. Finally, polymorphisms of the TNFα gene were correlated with a higher incidence of OAG in a glaucoma patient population [94,95]. Thus, the coexistence of a rise in TNFα expression and inflammatory changes clearly implicates TNFα in the pathogenesis of RGC loss in glaucoma.

## 4. Is Glaucoma a Neurodegenerative Disease?

As above-mentioned, IOP remains an important primary and prognostic risk factor for OAG and is used by clinicians to monitor anti-glaucoma treatments. However, despite controlled IOP, glaucomatous patients still continue to lose vision even after treatment [8], suggesting that independent mechanisms of IOP may contribute to progression of the disease. Additional data demonstrate that glaucomatous neuropathy spreads and impairs the central nervous system [6,7,8,9,10]. In a 12-month-old DBA/2J model, there was lower β-tubulin (a neuronal fiber marker) labeling in the optic nerve than in the control mice [96]. This suggests an anterograde deficit transport that can lead to damage to the first relays of visual information in the brain. After decussating, ON fibers divide into two central pathways. In rodents, the minor synaptic relay, which concerns only a small percentage (5%) of fibers, is the LGN. The major ON fiber relay (95%) in the rat brain is the superior colliculus (SC). Therefore, in an acute model of ocular hypertension (perfusion of the anterior chamber of eye with saline solution), an increase in the number of GFAP-positive astrocytes throughout the superficial layers of the contralateral SC was found [27]. In a model of laser photocoagulation of the perilimbal and episcleral veins, a significant enlargement of GFAP immunoreactivity was observed in both the contralateral and ipsilateral SC [97]. In the same preclinical unilateral model of glaucoma, we recently demonstrated that high IOP induces major neuroinflammatory responses (astrogliosis, microglia activation, and elevated pro-inflammatory cytokine expression) in the contralateral SC [44]. Finally, gliosis in the visual cortex has also been reported in nonhuman primates after photocoagulation of the trabeculum [98,99]. 

In glaucoma patients and primate models, over a period of IOP elevation, shrinkage and loss of neurons [11,12], reduced metabolic activity [13,14], and changes in the expression patterns of several synaptic plasticity markers [15] can be detected in the LGN and visual cortex. However, this ocular disease shares similar pathological mechanisms with those of neurodegenerative diseases such as Alzheimer’s and Parkinson’s. Deposits of amyloid β protein, synuclein, and pTau have been detected in the retinas of patients suffering from glaucoma. In addition, a recent proteomics study on the vitreous humor of glaucomatous patients found that 100 differentially regulated proteins are common to the pathophysiology of Alzheimer’s disease [100]. Like neurodegenerative diseases, glaucoma produces neuroinflammatory reactions, particularly with regard to the activation of glial and microglial cells [101]. Moreover, an increased release of pro-inflammatory cytokines (IL1, IL6, TNFα) and chemokines (CCL2, CX3CL1) at the first central relay level (LGN nuclei and SC) was detected. This leads to aggravation and propagation of the pathology to higher visual pathways. Several studies have shown that the apoptosis of RGCs is correlated with neurodegeneration of secondary neurons. More importantly, the activation of microglia, which not only occurs in hypertensive eyes, but also affects contralateral healthy eyes [25], proves the existence of complex ante- and retrograde neuroinflammatory mechanisms involved in the pathogenesis of glaucoma [44]. 

## 5. Neuropathologic Study of Neuronal Degeneration in the Visual Pathways

Neurodegenerative diseases share several fundamental characteristics including neuron loss specific to each pathology. The loss of these neuronal populations and the associated clinical impairment are correlated with the specific functions of these neurons. In glaucoma patients, visual field loss was correlated with increased neurodegeneration of RGCs [102]. The progressive loss of RGCs is therefore a characteristic of glaucoma pathology. However, what about the secondary neurons of the posterior visual pathway?

Lesions in the central visual pathway structures were observed in patients with advanced glaucoma. In histopathological studies on postmortem brain sections, lower neuronal density in the LGN, but also in the visual cortex of glaucomatous patients was found compared to the control [103,104]. A glaucoma model of macaque monkeys using laser photocoagulation of the trabeculum showed less labeling of synaptophysin (a synaptic protein) in the LGN, demonstrating lower connectivity [105]. This lower connectivity is correlated with LGN degeneration including shrinking of the cellular body [106] and neuronal death, leading to visual field alteration [99]. Indeed, quantitative evaluations of LGN neurons identified with parvalbumin labeling showed neuron degeneration in both magnocellular and parvocellular layers of the LGN [11,12] as well as cell body shrinkage. In murine models of ocular hypertension (using episcleral vein perfusion with saline solution), shrinkage and dendritic loss of neurons of the superior colliculus also occurred [107].

## 6. Neuroimaging Cerebral Anomalies in Glaucoma

In primates, 90% of RGCs convey visual information from the retina to the LGN [8]. Clinical data from glaucoma patients indicate that axon impairment extends from the optic nerve to the posterior optic pathways. In this context, it becomes relevant to investigate these changes by neuroimaging studies.

### 6.1. Magnetic Resonance Imaging

**Figure 2:**
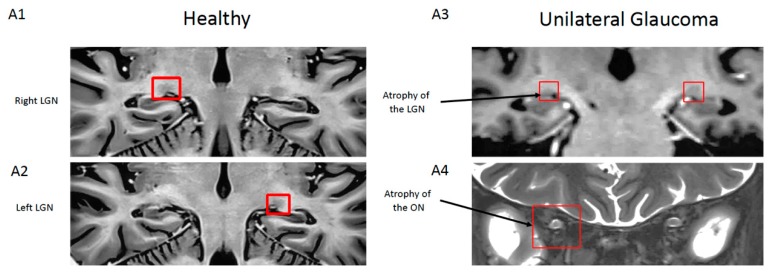
Magnetic resonance imaging (MRI) images of a coronal section of the brain of a healthy control and a unilateral glaucoma patient. Red squares localize the lateral geniculate nucleus (LGN) and optic nerve. MRI images from the patient show unilateral hyperintensity and atrophy of the right optic nerve (ON) and associated ipsilateral atrophy of the lateral geniculate nucleus compared to the healthy subject (from Dr. Habas Neuroimagerie XV-XX hospital; 3T SIEMENS Skyra MRI). (**A1**,**2**) Coronal contrast-inverted short-TI inversion-recovery (STIR) images of LGN. (**A3**) Coronal T1 weighted gradient-echo (MPRAGE) images of LGN. (**A4**) Coronal STIR image passing through the intraconal optic nerve.

A magnetic resonance imaging (MRI) study showed that all unilateral glaucoma patients exhibited bilateral involvement of their visual pathways, with the clinically symptomatic side predominating [108]. Much of the data seem to indicate that glaucomatous pathology for the most part results in retinal–thalamic visual pathway impairment. MRI analysis has made it possible to observe all of the secondary visual neurons. So, as previously mentioned, a relatively old histological study performed on postmortem tissues from glaucoma patients found lower neuronal density in LGN in patients than in the controls [103]. More recently, MRI studies confirmed this LGN atrophy in glaucoma patients (Figure 2) [109,110,111] as well as a thinner cortex of the visual pathways [112]. The diffusion–tensor imaging technique, which quantifies white matter abnormalities, was also used in glaucoma patients and demonstrated changes in white matter at the level of the optic radiations that project to the visual cortex [113,114,115]. MRI has also provided evidence of a decrease in gray matter density in the primary visual cortex in glaucoma patients compared to healthy subjects [116,117]. Finally, a resting state functional MRI study showed an alteration of functional connectivity between the primary visual cortex and the associative (secondary) visual areas [118]. These noninvasive neuroimaging and functional studies in glaucoma patients have shed light on the cerebral abnormalities of visual pathway structures such as atrophy, a loss of density, or an alteration of the communication between the visual areas.

### 6.2. Emission Tomography Imaging of Glial Activation

In vivo positron emission tomography (PET) imaging of glaucomatous primates (after laser trabeculoplasty) examined the binding of [11C] PK11195 (a ligand for the peripheral benzodiazepine receptor, PBR) as an index of the activation of glial cells in the lateral geniculate nucleus (LGN) [119]. This study showed that at all experimental stages of glaucoma including mild glaucoma, PET imaging at the LGN level was able to detect an increase in [11C] PK11195 binding density in all layers of LGN receiving the axons of retinal ganglion cell fibers of the injured eye. In addition, this increase in binding to PBR coincided with an increase in the immunostaining of microglia (Iba-1-ir) and astrocytes (GFAP-ir) observed on postmortem sections of LGN from the glaucomatous monkeys. Thus, these data suggest that PET using [11C] PK11195 as a ligand may be a useful noninvasive technique for the diagnosis of glaucoma.

## 7. Conclusions

The current management of glaucoma focuses on treatments to reduce intraocular pressure and remains the main pharmaceutical strategy today. However, despite effective IOP control, these therapeutic approaches will ultimately prove ineffective in preventing worsening of the condition and long-term visual loss. Numerous preclinical and clinical data relating to glaucoma tend to show that neuroimmune and neurodegenerative events continue to occur in the retina and spread to the visual areas of the brain [3,18,120]. Thus, a detailed spatio-temporal knowledge of these pathological events is today crucial for the development of new therapeutic strategies with immunomodulatory and/or anti-apoptotic aims to prevent blindness in glaucoma. Currently, new drugs are being tested in the management of glaucoma such as the effects of cannabinoids on neuroprotection and inflammation [121], cellular therapies based on stem cells or their secretome to immunomodulatory and neuroprotective purposes [122] and the use of antibiotics with immunomodulatory properties [123]. However, considerable clinical and basic research must continue to be carried out to refine these new therapeutic approaches against glaucoma pathology. Therewith, the development of more accurate neuroimaging techniques could be useful to develop a detailed representation of the impact of these events on the visual pathways and their evolution over time and appreciate the therapeutic potential of new anti-glaucoma strategies.
